# Viruses and Cancer

**Published:** 1966-07

**Authors:** Edward S. Meek

**Affiliations:** University of Bristol Medical School


### VIRUSES AND CANCER

BY

EDWARD S. MEEK

*University of Bristol Medical School*

Three types of agent cause cancer — radiation, chemicals and viruses.

The first demonstration of induction of cancer by a biological agent was made in 1911 by Rous who transmitted a tumour of fowls from one bird to another by means of cell-free extracts; later work has shown that such extracts contain the organism now known as the Rous sarcoma virus. More recently, a number of other avian tumours, including leukoses, erythroblastosis, sarcomata and renal tumours, have been found to be of viral origin. For many years it was thought that the phenomenon of tumour induction by virus was a peculiarity of birds but this view has since been disproved. There is abundant evidence that in mammals, and amphibia also, viruses are a causal factor in the development of some neoplasms.

One well-known example is that of mammary carcinoma of mice infected with the Bittner virus. At present, eight different viruses are known which can give rise to leukæmia and lymphosarcoma in mice. Rats may also develop leukæmia through viral action, and virus-transmitted leukæmia has been reported in guinea-pigs; in rabbits, both papillomas and fibromas can be induced by oncogenic viruses. A virus-promoted kidney tumour is known in frogs.

Without compiling an exhaustive list, it is seen that a variety of neoplasms occur in amphibia, birds and mammals as a result of virus infection, and it is difficult to believe that man is an exception to this pattern. So far, however, no malignant human tumours have been shown to be caused by a virus, and our knowledge of the characteristics of oncogenic viruses rests on observations of animal and bird tumours. What is known about them?

In the first place, the idea that each specific type of virus invokes only one histological type of tumour does not appear to be true in all cases. The polyoma virus of mice is capable of promoting the development of a number of different primary neoplasms including parotid gland tumours, leukæmia, hæmangioma, thymomas (both epithelial and lymphocytic), nephroblastoma, fibrosarcoma and osteosarcoma; sometimes two or three different types of primary tumour arise in the same animal simultaneously. The fowl tumour viruses appear to form a group of very closely related strains; some investigators have recently purified some of these so as to yield a higher incidence of a particular neoplasm. The effect of cell susceptibility is tied up with this, however, and the response of various tissues to a given virus may depend partly on the age and development of the host. Rous sarcoma virus, if given to a chick in the early stages of growth, does not give rise to a tumour at all but leads to non-neoplastic malformations of blood vessels.

Not only may some of these viruses yield more than one type of tumour, but some may also manifest their oncogenic properties in more than one species. Rous sarcoma virus, originally isolated from fowls, can produce tumours not only in ducks and turkeys, but also in rats and hamsters; Rabotti, Raine and Sellers (1965) report the formation of gliomas in the latter. Polyoma virus can give rise to neoplasms in mice, rats, hamsters, guinea pigs and rabbits, while the Lucké renal tumour virus of frogs will induce cartilaginous tumours in newts. Nearer to the human problem is the simian vacuolating virus 40 (SV-40) which is found in a high proportion of rhesus monkeys and, until 1962, was probably present in many batches of the vaccine produced in cultures of monkey tissues for immunisation against poliomyelitis; this agent will cause tumour formation in hamsters upon injection. Since some of these viruses may be widespread in a wild population of natural hosts without apparently causing ill effects, the use of tissue cultures for the production of vaccines calls for careful consideration as the oncogenic activity of latent viruses may only become apparent after introduction into other species.

A puzzling feature of viral oncogenesis is that a considerable time may elapse between the time of infection and the appearance of cancer. For instance, injection of virus into newborn mice results in the development of leukæmia in a high percentage of the animals but not until the age of one year, a period corresponding to some twenty or thirty years in man. In the case of the Bittner mammary cancer of mice, although the animals are infected in the first few days of life during suckling, tumours do not develop until after litter-bearing in adult life; in this case, the expression of viral action has been shown to depend on the presence of a high level of œstrogen — such as that associated with pregnancy — and may be affected still further by genetic factors of resistance or susceptibility. It would seem, then, that the mere presence of oncogenic virus may not be enough to produce cancer; host-controlled factors may modify its activity.

Turning now to human investigations, it has been shown that injections of human adenovirus into hamsters and mice can lead to the development of malignant neoplasms. Several of the thirty-one known types have this property, and the adenoviruses, which cause respiratory infections in man, are widespread amongst the population according to recent surveys of serum antibody distribution.

In view of the number of leukæmogenic viruses now isolated from other species, interest in the possible viral ætiology of human leukæmia is high. Heath and Hasterlik (1963) reported an unusual number and grouping of cases of leukæmia in children near Chicago, and drew attention to the resemblance to a mild localised epidemic. In 1964, Negroni and his colleagues at the National Institute for Medical Research, London, found virus-like particles present in cultures of marrow from cases of leukæmia (Inman *et al.*, 1964). These have since been identified not as viruses but as mycoplasma, an organism resembling bacteria. However, since some types of mycoplasma appear to be commensal organisms in some parts of the body, there is doubt as to whether these play any causal role in leukæmia.

Burkitt (1958) has described a form of malignant lymphoma common in parts of Africa, which is manifest by development of tumours in bone, thyroid, kidneys, ovaries, heart and other organs. This condition is confined mainly to children between the ages of three and twelve years, is independent of tribe or race, and has a peculiarly limited geographical distribution. Burkitt (1963) has suggested that this illness is due to a virus spread by an insect vector; so far, there is no definite evidence showing a specific virus to be the causal agent, but the search is being continued energetically.*

Numerous attempts have been made to isolate viruses from human tumours by injection of neoplastic material into experimental animals or cell cultures, but the results have been negative or equivocal. Can it be that virus is present in a masked form and not amenable to the usual methods of extraction? There is evidence to suggest that this could occur. Whilst the tumour cells initiated by avian viruses normally continue to produce and release large quantities of virus, it has been shown that some normal chick cells can be transformed by virus into malignant cells which fail to produce any more virus. The suggestion that the continued production of virus is unnecessary for the growth of tumours, is supported by work with other oncogenic viruses. It has been found that many tumours induced *in vivo* by injection of virus (polyoma, adenovirus, SV-40) fail to produce or release virus once the tumour growth is well established. Further study has yielded indirect evidence, however, that some portion of the virus persists within the cells throughout numerous cell divisions and apparently multiplies in step with the host cells. The portion which persists is evidently viral genetic material (nucleic acid) which is thought to be integrated with that of the host. This would explain the long-continued production of virus-induced antigens within the cells in the apparent absence of whole virus particles.

The part played by antibodies against these viruses calls for consideration. Antibodies directed against the virus particles themselves can be found, often in very high titre, in animals bearing these tumours. In the circumstances, the antibody presumably inactivates and prevents the spread of virus from one cell to another through the body fluids; however, it appears incapable of reaching the virus within the cells. Following

* Since this was written, new evidence has pointed to two likely candidates—reovirus type 3, and the virus of herpes simplex.

the initial infection, there is a lag period before antibodies appear; if virus can establish itself firmly within the cells during this period, it would be immune thereafter to the antibody response, though unable to spread further.

The position is complicated, however, by the fact that the cells containing this ' integrated ' virus now develop a new cellular antigen within the nucleus. Unfortunately, this new nuclear antigen seems to elicit only a very weak antibody reaction. If it should prove possible to enhance this reaction, then it may lead to elimination of cells bearing integrated material from oncogenic virus.

These findings suggest that the straightforward methods for isolation of tumour-viruses from human neoplasms may be inadequate. Before any headway can be made two major problems have to be solved; one is the identification of viral genetic material in tumour cells which fail to yield whole virus particles after homogenisation. The second is to activate this genetic material in such a way that production of intact virus particles is resumed; some progress has been made in this direction with avian and animal tumours where integrated virus has been induced to proliferate by the action of radiation and chemicals. It is likely that more sophisticated attempts to isolate virus from human tumours will be made, and if successful would permit their culture *in vitro* for further study and characterisation.

In conclusion, the viruses we seek may be widely distributed throughout the population though inactive in the majority of carriers. They may be acquired by infection early in life, but then remain quiescent for many years or even a lifetime. Alternatively, they may be activated after a long latent period by changes in the host environment; these changes may include alterations in hormone levels or result from the inhalation or ingestion of " carcinogenic " chemicals. Finally, oncogenic viruses may be present in tumour cells in a masked form and not available for isolation by present methods.

### REFERENCES

Burkitt, D. (1958). "A Sarcoma Involving the Jaws in African Children". *Brit. J. Surg.*, **46**, 218.

Burkitt, D. (1963). "A Children's Cancer Dependent on Environment". In "Viruses, Nucleic Acids and Cancer" (Williams and Wilkins, Baltimore), p.615 *et seq.*

Heath, C. W. and Hasterlik, R. J. (1963). "Leukaemia among Children in a Suburban Community". *Amer. J. Med.*, **34**, 796.

Inman, D. R., Woods, D. A. and Negroni, G. (1964). "Electron Microscopy of Virus Particles in Cell Cultures Inoculated with Passage Fluid from Human Leukaemia Bone Marrow". *Brit. Med. J.*, **1**, 927.

Rabotti, G. F., Raine, W. A. and Sellers, R. L. (1965). "Brain Tumors (Gliomas) Induced in Hamsters by Bryan's Strain of Rous Sarcoma Virus". *Science*, **147**, 504.

Rous, P. (1911). "Sarcoma of the Fowl Transmissible by an Agent Separable from Tumor Cells". *J. Exp. Med.*, **13**, 397.

